# Improved Tumor Targeting and Longer Retention Time of NIR Fluorescent Probes Using Bioorthogonal Chemistry: Erratum

**DOI:** 10.7150/thno.128809

**Published:** 2026-01-30

**Authors:** Xianghan Zhang, Bo Wang, Na Zhao, Zuhong Tian, Yunpeng Dai, Yongzhan Nie, Jie Tian, Zhongliang Wang, Xiaoyuan Chen

**Affiliations:** 1 Engineering Research Center of Molecular-imaging and Neuro-imaging of ministry of education, School of Life Science and Technology, Xidian University, Xi'an, Shaanxi 710026, China;; 2 Institute of Automation, Chinese Academy of Sciences, Beijing 100190, China;; 3 Institute of Digestive Diseases, Xijing Hospital, Fourth Military Medical University, Xi'an, Shaanxi 710032, China;; 4 Laboratory of Molecular Imaging and Nanomedicine, National Institute of Biomedical Imaging and Bioengineering, National Institutes of Health, Bethesda, Maryland, 20892 USA.

In the original version of the Supplementary Material, the H&E graph in Figure S10 was misplaced during data organization. The correct version of Figure S10 is shown below. The correction made in this erratum does not affect the original data and conclusions. The authors apologize for any inconvenience that the errors may have caused.

## Figures and Tables

**Figure A:**
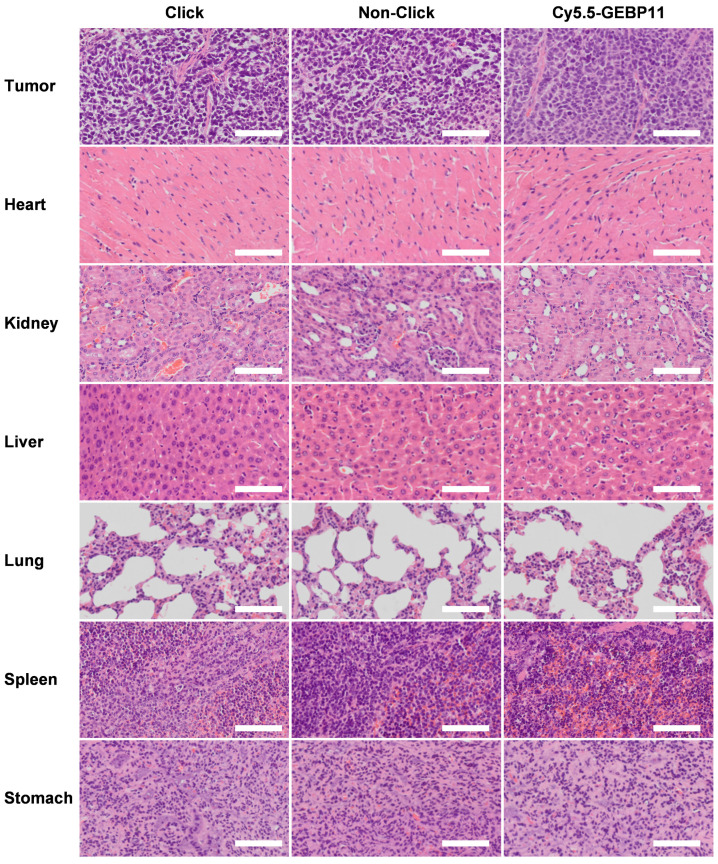
** Corrected Figure S10.** Hematoxylin and eosin (H&E) pathology examinations. The heart, kidney, liver, lung, spleen and stomach of click, non-click, Cy5.5-GEBP11 groups did not show obvious structural changes. The scale bar represents 100 *μ*m.

